# Progressive Dwindling in Multiple Sclerosis: An Opportunity to Improve Care

**DOI:** 10.1371/journal.pone.0159210

**Published:** 2016-07-21

**Authors:** Jessica E. Martin, Joel Raffel, Richard Nicholas

**Affiliations:** Centre for Neuroinflammation and Neurodegeneration, Division of Brain Sciences, Department of Medicine, Imperial College London, London, United Kingdom; Universitá Cattolica del S. Cuore, ITALY

## Abstract

**Introduction:**

In the general ageing population, 40% of deaths occur following a prolonged trajectory of “progressive dwindling,” characterised by chronic accumulation of disability and frailty, and associated with increased dependency and reduced reserves. Those who progressively dwindle are poorly catered for by current healthcare systems and would benefit from a coordinated approach to their medical and social care, known as formative care. People with multiple sclerosis (pwMS) may be more likely to progressively dwindle, and may be appropriate targets for formative care pathways.

**Objectives:**

To determine the proportion of pwMS who follow a progressive dwindling trajectory prior to death. To relate trajectory to place of death, and examine what factors predict the progressively dwindling trajectory.

**Methods:**

A retrospective observational study of 582 deceased pwMS enrolled in the UK MS Tissue Bank, including death certificates and extensive clinical summaries.

**Results:**

73.7% of pwMS had a “progressively dwindling” trajectory of dying. This was predicted by those who reach MS disease milestones earlier. 72.5% of pwMS died an MS-related death, which was predicted by an aggressive disease course from onset. Those who progressively dwindled were equally likely to die in hospital as those with other trajectories to death.

**Conclusions:**

The progressively dwindling trajectory of dying is very common in pwMS, and can be predicted by earlier disease milestones. Pathways could target pwMS in these years prior to death, to improve care.

## Introduction

Over the last century there has been a shift in the commonest causes of death, from acute causes at a young age, to a substantially lengthened lifespan characterised by a period of progressive chronic illness before death. [1] Bowman and Meyer (2014) described four broad trajectories of dying in an ageing population: [2]

- 20% are sudden e.g. myocardial infarction;
- 20% of deaths follow a short period of rapid decline after a ‘clear clinical transition’ from treatable to progressive e.g. cancer;
- 20% occur as a result of acute exacerbations of a progressive long-term disease e.g. chronic obstructive pulmonary disease;
- 40% of deaths occur following a prolonged period of ‘progressive dwindling’ e.g. Alzheimer’s disease.

For the first three trajectories, healthcare infrastructure is well established and effective, with preventative and emergency medicine for the sudden trajectory, palliative care for those with a short period of rapid decline, and specialist chronic disease management programmes for those with progressive long-term diseases with acute exacerbations. [2] However, there is a lack of appropriate pathways for those who ‘progressively dwindle’, with often haphazard involvement of a wide range of healthcare professionals and services, and thus care can be sub-optimal. [2] A report from the UK Parliamentary and Health Service Ombudsman in May 2015 found a lack of coordination of care services, delays in referral and inadequate out-of-hours services have led to poor end of life care for a large number of patients. [3] The report suggested this could be improved by proper service delivery and organisation. [3] The term ‘formative care’ has been coined to describe the strategy of “enabling the best possible life quality and experience in the context of a life reframed by frailty and dependency” for progressively dwindling patients in the time between active treatment and end of life care. [2] Aspects of formative care overlap with palliative care, including symptom relief and home care, although formative care focuses on maximising quality of life rather than quality of death, and takes place over years rather than weeks or months. Elements of formative care might include access to regular tailored physiotherapy, occupational therapy visits to the home environment, nutritional support, pathways to determine when packages of care should be escalated and improving this transition, and rationalising medication, focusing on those that improve quality of life. Promoting implementation of formative care from an early stage will also allow improved transition into palliative care, and act as a buffer against difficulties in determining when palliative care should begin. Identifying markers to predict those who will adopt a progressive dwindling trajectory will be essential for implementing formative care in a timely manner for these patients.

People with MS (pwMS) have a prolonged disease course, often 50 years or more. [4] Disease modifying therapies are available for relapsing MS, but there are currently no licensed treatments for the progressive phase. As a result, a gap in structured care pathways has arisen between active treatment and end of life care, in which pwMS may spend a prolonged period ‘progressively dwindling’, with considerable distress associated with decline in physical and mental function. [5] This period without active treatment but before palliation is not specific to MS, but is particularly pertinent due to the extended and unpredictable timeframe. It is during this phase that pwMS could greatly benefit from formative care. Those pwMS who progressively dwindle experience a slow and steady accumulation of disability, resulting in frailty, and associated with increased dependency and reduced physiologic reserve. [6] This period extends for many years before death, during which time there is an increasing burden of care, often taken on by the family, and punctuated by superimposed illness. As the ever-reducing physiologic reserve resets the baseline, there is reduced capacity to cope with said periods of superimposed illness, such as pneumonia, and ultimately these present the terminal event. [6] Because formative care promotes adequate planning of and improved transition to palliative care due to overlap in techniques, it should also empower patients to choose to die at home, if this is their wish. To understand how formative care might help pwMS, this study aimed to: (i) determine the frequency of progressive dwindling in an MS population, (ii) identify factors associated with progressive dwindling, (iii) determine specific cause of death and whether deaths were ultimately MS-related or not, and (iv) examine place of death in those with different trajectories to death.

## Materials and Methods

### Data collection

Data were retrospectively collated from a cohort of 582 pwMS who had died between January 1998 and February 2015 inclusive, and had been registered on the UK MS Tissue Bank (UKMSTB). The UKMSTB is a national scheme to collect post-mortem tissue donated from pwMS and non-MS controls, as well as death certificate data, clinical summaries and extensive clinical notes. As of February 2015, the UKMSTB stored post-mortem data on 606 pwMS; all were included in this study after exclusion of 9 donors without a confirmed tissue diagnosis of MS, and 15 donors with insufficient data on cause of death. Clinical notes were used to define the date of symptom onset, progressive disease (defined as a period of at least one year with gradual disability worsening without relapses), wheelchair use, and death, for all patients. Participants with missing data on symptom onset, progression, wheelchair use, and/or place of death were included in the study, but were excluded from relevant analyses. The UKMSTB has ethical approval from the London Multicenter Research Ethics Committee (MREC/02/2/39) to prospectively recruit donors after obtaining written informed consent, and accrue an extensive clinical summary from medical notes and death certificates as previously described. [7]

### Classification of death: Cause, MS-related, Trajectory, and Place

Death certificate entries were used to categorise underlying cause of death (UCD), and confirmed using patient notes and clinical summaries when the death certificate was unclear. For example, if the death certificate stated ‘infection’ or ‘respiratory failure’, clinical notes were used to identify a more specific UCD e.g. pneumonia. If multiple causes were listed on the death certificate, clinical notes were used to discern the primary cause of death. If multiple causes of death were listed, the diagnosis that triggered the chain of events leading to death was chosen (e.g. pneumonia leading to respiratory failure). Where it could not be discerned whether one was more responsible that the other (e.g. pneumonia and UTI), and the clinical notes did not clarify, the first listed was used as the UCD. UCD was only categorised as MS when no other cause was listed; where possible a more specific cause (e.g. pneumonia) was used, even if secondary to MS. Deaths were also classified as being ‘related to MS’ or not, by examining death certificates and clinical notes. Trajectory to death categories were adapted from Bowman and Meyer; [2] trajectory was based on cause of death and review of the clinical summaries. Place of death categories were based on Public Health England’s place of death classification. [8] Own residence, care home and hospice deaths were also combined into a ‘community’ category.

### Statistical analysis

Cox proportional hazards regression was performed to compare survival from disease milestones between different groups e.g. those with MS-related deaths vs. those with non-MS-related deaths, those who progressively dwindled vs. those with other disease trajectories. Disease milestones were compared between all four disease trajectory groups using one way between subject ANOVA, with Tukey HSD used post hoc for significant ANOVA analyses. Student’s t-test was used to compare demographics in males and females. Parametric tests were performed after testing for normality. Chi-squared test was used to compare location of death in those with different trajectories to death, and those with and without MS-related deaths. Yates’ chi-squared test was used to avoid overestimation of statistical significance. Where chi-squared revealed significance in contingency tables greater than 2x2, further post hoc 2x2 chi-squared tests were carried out to identify the significant values, with criteria for statistical significance adjusted according to the number of post hoc tests carried out.

## Results

### Study population and cause of death

582 deceased pwMS were included in the analysis. 70% were female, and the average age at symptom onset was later among females than males (34.1±10.7 [mean±SD] vs 30.6±9.7 p<0.001). The average age of progression was 45.5±11.3, the average age of wheelchair use was 50.6±12.8 and the average age at death was 63.8±12.7 years with women dying later than men (64.9±13.1 vs 61.2±11.5, p<0.01). 68.5% were categorised as secondary progressive MS at the time of death, 11.4% primary progressive, 6.0% relapsing remitting, and the sub-type of MS was unavailable in 14.1%. The most common UCD was pneumonia or bronchopneumonia (37.5%), followed by MS (14.8%), cancer (10.1%), and aspiration pneumonia (8.6%; Table 1). 72.5% died an MS-related death. 35.9% of pwMS did not have MS recorded on any part of their death certificate; including 28.7% of those whose death was adjudged to be MS-related after inspection of clinical notes in this study.

**Table 1:** Cause of death of 582 people with MS, UKMSTB January 1998 to February 2015.

Cause of death specific categories	n (%)
Multiple sclerosis	86 (14.8)
Pneumonia, bronchopneumonia	218 (37.5)
Aspiration pneumonia	50 (8.6)
Urinary tract infection	34 (5.8)
Other infection, inc. sepsis	12 (2.1)
Acute cardiovascular event	22 (3.8)
Acute cerebrovascular event	18 (3.1)
Pulmonary embolism	14 (2.4)
Non-acute cardiac e.g. senile myocardium or heart failure	11 (1.9)
Respiratory failure	9 (1.5)
Infection secondary to chronic obstructive pulmonary disease	7 (1.2)
Cancer	59 (10.1)
Acute abdomen e.g. obstruction	13 (2.2)
Suicide	7 (1.2)
Epilepsy	3 (0.5)
Other e.g. fulminant liver failure, renal failure, dehydration, acute pyelonephritis, tuberculosis, old age, myelodysplastic syndrome, liver abscess, progressive multifocal leukoencephalopathy, general deterioration, anaphylactic reaction, cardiac asthenia amyloid, ruptured left subclavian artery aneurysm, necrotic fasciitis, accidents, dementia, and frailty.	19 (3.1)

### MS-related deaths are predicted by markers of aggressive disease

**Fig 1:**
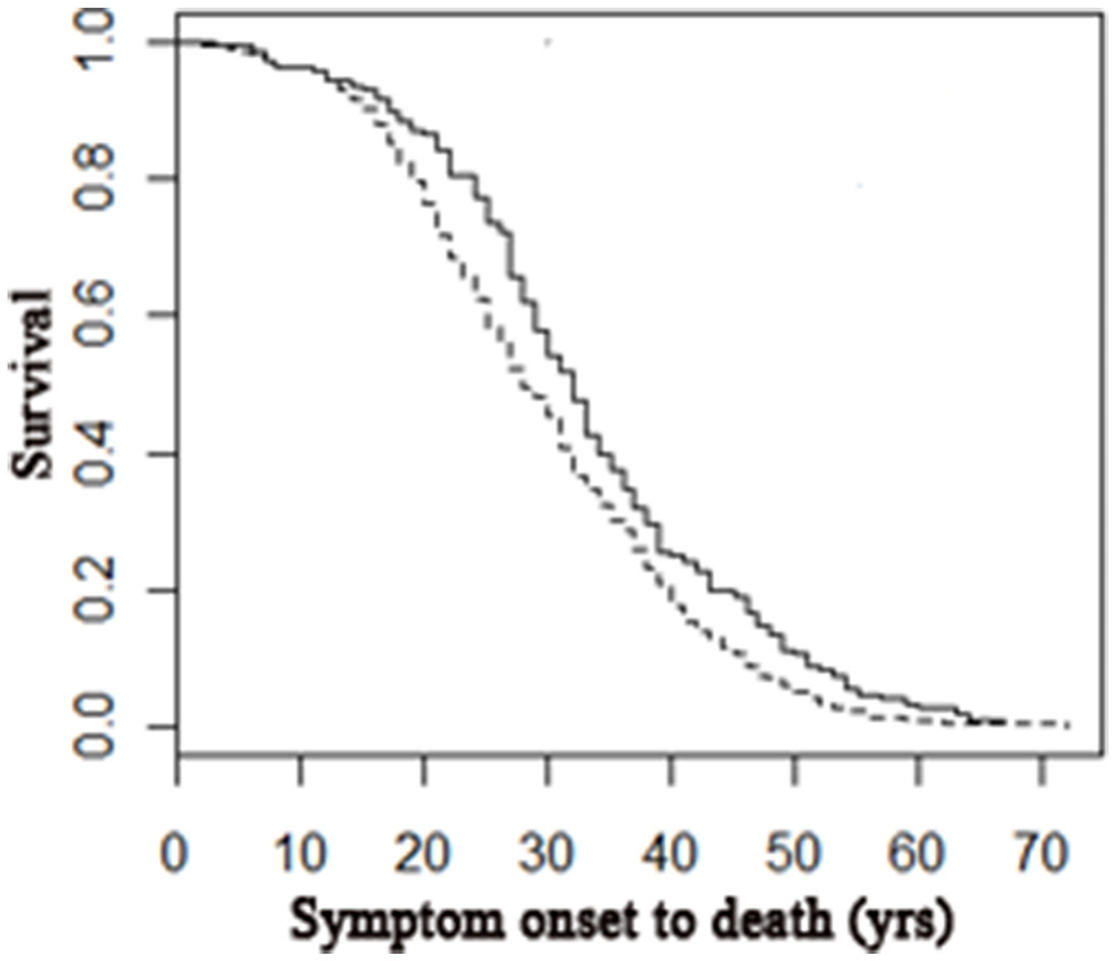
Cox proportional hazard regression model comparing disease length leading to MS-related deaths and unrelated deaths. Those whose death was MS-related had a shorter disease course from symptom onset to death than those whose death was unrelated (R^2^ = 0.016, n = 504, p<0.01; MS death 1.32, 95CI 1.08–1.61). Dotted line represents MS-related deaths; solid line represents non-MS-related deaths.

Of the 582 pwMS, 72.5% died an MS-related death. MS-related deaths were associated with younger age at symptom onset (MS-related: 31.6±9.9; non MS-related 36.9±10.9, p<0.0001), progression (MS-related: 43.5±10.3; non MS-related 51.3±11.9, p<0.0001), wheelchair use (MS-related: 48.4±11.9; non MS-related 57.6±12.9, p<0.0001) and death (MS-related: 61.5±12.4; non MS-related 68.9±11.6, p<0.0001). MS-related deaths were also associated with a shorter time from symptom onset to death (MS-related: 29.4 ±11.9; non MS-related 32.9±12.7, p<0.01). These earlier milestones are indicative of more aggressive disease courses leading to MS-related deaths (Fig 1).

### Progressive dwindling is a common trajectory in MS, and is predicted by earlier disease milestones

**Fig 2:**
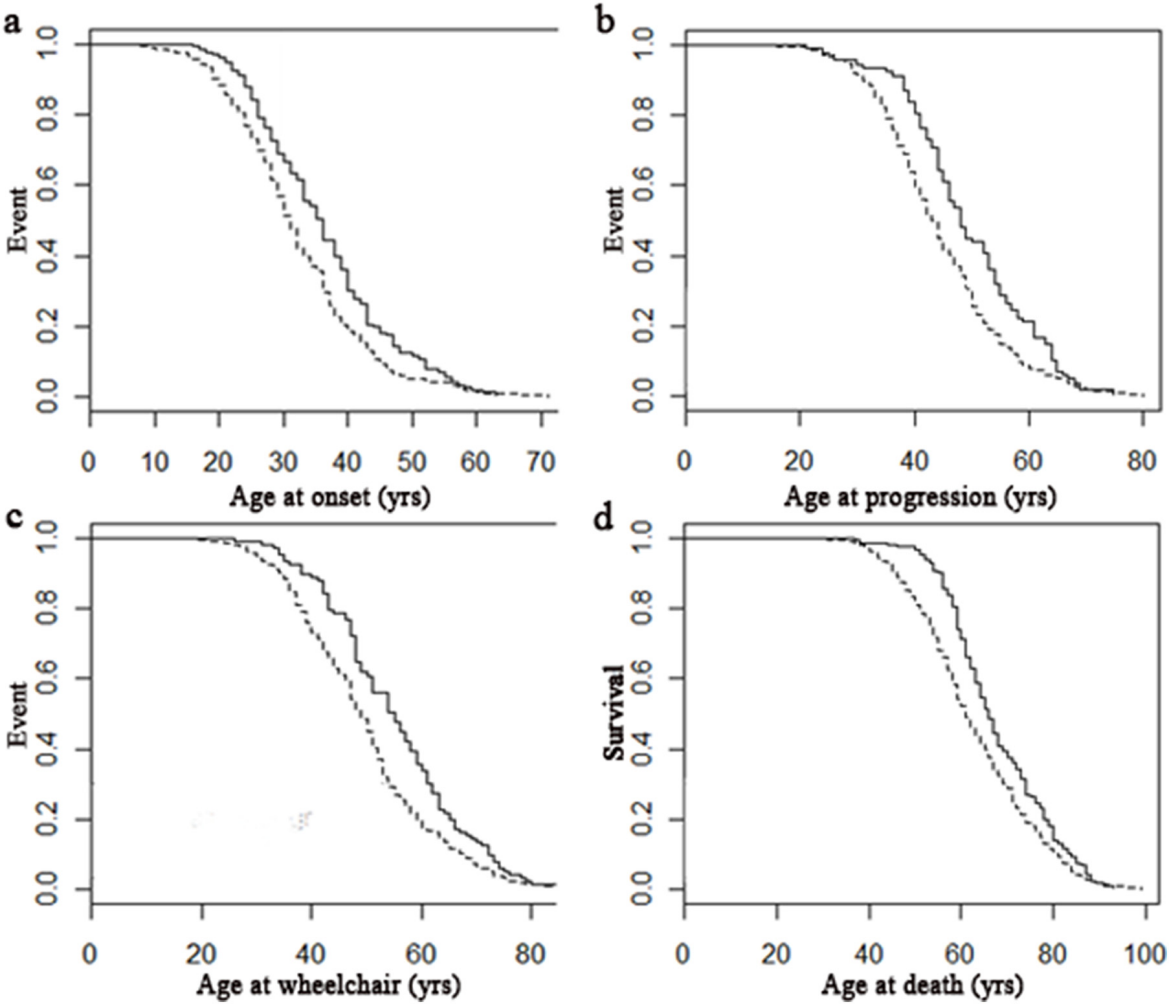
Cox proportional hazard regression models of disease milestones in those who progressively dwindle compared to other trajectories. Those who progressively dwindle had an earlier age at onset (A, R^2^ = 0.02, n = 504, p<0.01; Progressive dwindling: 1.08, 95CI 1.03–1.14), progression (B, R^2^ = 0.024, n = 390, p<0.01; Progressive dwindling: 1.09, 95CI 1.03–1.16), wheelchair use (C, R^2^ = 0.022, n = 462, p<0.01; Progressive dwindling: 1.09, 95CI 1.03–1.15) and death (D, R^2^ = 0.014, n = 582, p<0.01; Progressive dwindling: 1.07, 95CI 1.02–1.12). Dotted lines represent those with progressive dwindling trajectory to death; solid line represents all other disease trajectories.

Of 582 pwMS, 429 (73.7%) progressively dwindled, 76 died a sudden death, 59 experienced a clear clinical transition from a treatable to an unrelenting progressive disease (e.g. cancer), and 18 died as a result of an acute exacerbation of a progressive long-term condition. When comparing those who progressively dwindled with the other trajectories combined, those who progressively dwindled had earlier age at onset, progression, wheelchair use and death (all p<0.01; Fig 2). Differences between groups remained when comparing individual groups in a one-way between subjects ANOVA (Table 2). More specifically, after post-hoc Tukey HSD, those who progressively dwindled had a mean age at MS onset five years earlier than those who died suddenly (p<0.01). Those who experienced a progressive dwindling trajectory had an earlier age at progression than those who died following a clear clinical transition (p<0.05) or an acute exacerbation (p<0.01). Those who experienced a progressive dwindling course were wheelchair-bound 9 years earlier than those who died following acute exacerbations and 5 years earlier than those who died suddenly (both p<0.05). Those who progressively dwindled died at a younger age than those who experienced any other trajectory to death (all p<0.05). There was no significant relationship between the trajectory to death and interval from symptom onset to death.

**Table 2:** Different Trajectories association with MS milestones.

Trajectories	Mean age at symptom onset ±SD (n)	Mean age at progression ±SD (n)	Mean age at wheelchair ±SD (n)	Mean age at death ±SD (n)
**Clear clinical transition**	35.3±10.0 (54)	50.2 ±9.9 (38)	54.2±10.6 (39)	67.7±10.2 (59)
**Acute exacerbation of progressive long-term condition**	35.5±10.4 (15)	54.5±15.3 (12)	58.5±13.6 (13)	71.2±9.2 (18)
**Sudden**	36.9±10.7 (65)	47.1±10.4 (42)	54.4±13.0 (56)	66.5±12.1 (76)
**Progressive dwindling**	32.0±10.3 (370)	44.3±11.1 (298)	49.3±12.6 (354)	62.5±13.0 (429)
**ANOVA Significance**	p<0.01^a^	p<0.001^a^	p<0.001^a^	p<0.001^a^

^a^The significant associations as identified by post-hoc Tukey HSD are detailed under ‘Progressive dwindling is a common trajectory in MS, and is predicted by earlier disease milestones’.

### Location of death

Of 582 donors included in the study, place of death was available for 503 donors (86.4%). Of those, 50.7% died in hospital, 25.4% died in a care home, 18.9% died in their own residence, 4.6% died in a hospice and 2 died in other locations. Those who progressively dwindled were equally likely to die in hospital as those with other trajectories to death (Table 3).

**Table 3:** Community vs. hospital and trajectory to death.

	Community n (%)^a^	Hospital n (%)	Total
**1 Clear clinical transition**	33 (61.1)	21 (38.9)	54
**2 Acute exacerbations of progressive long-term conditions**	3 (23.1)	10 (76.9)	13
**3 Sudden**	32 (47.8)	35 (52.2)	67
**4 Progressive dwindling**	178 (48.5)	189 (51.5)	367
**Total**	246 (49.1)	255 (50.9)	501^b^

^a^Own residence, care home and hospice deaths were combined into a ‘community’ category.

^b^Two died in ‘other’ locations which were not classified into community or hospital.

Similarly, those whose death was MS-related were equally likely to die in hospital as those whose death was unrelated to MS (Table 4). Those with MS-related deaths were less likely to die in a hospice—a category dominated by those with cancer-related deaths. Dying in the community rather than in hospital was not associated with an earlier death or a shorter interval from symptom onset to death.

**Table 4:** The relationship between place of death and whether a death was MS-related.

	MS-related death n (%)	Not MS-related death n (%)
1 Own residence	67 (70.5)	28 (29.5)
2 Hospital	184 (72.2)	71 (27.8)
3 Care homes	98 (76.6)	30 (23.4)
4 Hospice^a^	9 (39.1)	14 (60.9)
5 Other^b^	0	2
Total	358 (71.2)	145 (28.8)

^a^Significantly lower numbers of subjects died an MS-related death in hospices compared to those who had a non MS-related death (p<0.01, χ² test).

^b^Excluded from χ² test

## Discussion

This study finds rates of progressive dwindling in pwMS far greater than in the general population. These results emphasise the extended and unpredictable timeframe between active treatment and end of life care in MS, and the high proportion of pwMS who have prolonged periods of frailty, dependency, and reduced physiological reserve for many years prior to death. Those who progressively dwindle are equally likely to die in hospital as those with more acute trajectories to death, which might be interpreted as a failure of care. This study also finds that progressive dwindling can be predicted by early markers of aggressive disease in pwMS, suggesting an opportunity for targeted formative care in this subset of patients.

The UKMSTB is a community based scheme where people with MS register during their lifetime for tissue and clinical data to be collected when they die. Donors are representative of MS patients nationwide as a result of a national community-based recruitment strategy, with accurate representation of clinical milestones and MS sub-types confirmed by comparison with other study populations. [7] One advantage of this large cohort of 582 pwMS is that extensive clinical summaries are available for each donor, to allow accurate categorisation of disease milestones, trajectories, and cause of death. In addition, it was possible to include cases regardless of whether MS was mentioned on the death certificate, in contrast to previous studies on mortality in MS. [9, 10] Indeed, in our cohort, MS was not mentioned on the death certificate in 35.9% of cases, many of which we adjudged to have died an MS-related death after inspection of clinical records. Furthermore, by manually categorising UCD in each patient, we mitigated the effect of heterogeneous death certification technique and coding rule changes which can lead to inconsistent reporting of UCD within other studies. [10–12]

The most important limitation of this study was that data were retrospectively collated from clinical records and notes. The required data was not apparent on occasion, for example the ‘date of wheelchair use’ may not have been documented, while on other occasions coding of data required interpretation by the assessor. However, care was taken to formulate standardised rules for coding, and the availability of clinical notes improved reliability of data over death certificates. Overall, manual determination of these factors was considered to be a strength of this study as it allowed a more thorough analysis of each case, using the clinical summary where necessary. Another possible limitation is that cases were included whose deaths occurred between 1998 and 2015. Standards of care may have changed over this period, although the results reported in this study appeared consistent over time.

The most common cause of death was pneumonia (46%), followed by MS (14.8%), and cancer (10.1%). The rates of pneumonia and MS deaths are different to those found in other studies, [12, 13] however this is due to methodological differences; UCD was only classed as MS where no other cause was available, in order to allow more specific analysis of cause of death. Diseases of the respiratory system accounted for 15% of deaths in the UK in 2013 and therefore are far more common in our MS cohort. [14] Cancer is the most common cause of death among the general population (29%) followed by diseases of the circulatory system (28%); both much higher rates than in our cohort. [14] Accidents and suicide accounted for 1.2% of deaths in our sample, equal to rates in the UK general population in 2013. [14, 15] Although only 14.8% of death certificates directly attributed UCD to MS, 72.5% of deaths were MS-related. Younger age at symptom onset, wheelchair use, progression and death, as well as a shorter disease course, were all associated with MS-related death. This earlier achievement of disease milestones and shorter interval between milestones is suggestive of a more aggressive disease course; those who evade this aggressive disease course are more prone to the same causes of death as the general population.

This study found that 73.7% of pwMS had a progressively dwindling trajectory prior to death. This is in contrast to the general ageing population, in which 40% of the population progressively dwindle. [2] Those who progressively dwindled experienced an earlier age at symptom onset, progression, wheelchair use, and death than those experiencing other trajectories, indicating that earlier disease milestones predict progressive dwindling.

In 2006, 58% of general population deaths in the UK were in hospital and only 35% of people died at home or in a care home, despite 56–74% of people saying they would prefer to die at home. [16, 17] As a result of this majority preference for dying at home, place of death is often considered a surrogate marker of the success of end of life care. Of 503 pwMS in this study, 50.8% died in hospital, 25.4% died in a care home, 18.9% died in their own residence, and 4.5% died in a hospice, consistent with previous work. [10] Those who died following a progressive dwindling trajectory were as likely to die in hospital as any other group, a statistic that might be improved with the implementation of formative care pathways for this group, as the nature of the trajectory allows time to discuss patient preferences and establish appropriate plans. Major barriers to dying in the community include poor provision of end of life care services by general practices, insufficient community nursing staff, poor coordination of services, and lack of access to home modifications and out-of-hours medicines. [18] MS patients may be more vulnerable to these service deficiencies, as a result of their comparably young age, complex care needs and increased rates of cognitive disability. [19] Implementation of a structured package of formative care, with discussions at various stages of the process, would give pwMS opportunities from an early stage, to communicate how they wish to live, and die, as well as providing a more suitable and coordinated care network to meet their requirements.

As people approach death, an increasing number of pwMS and their caregivers desire support from palliative care services either at home or in hospices. [17, 20, 21] In our study, those who died an MS-related death were far less likely to die in a hospice than those who died a non-MS related death, suggesting hospice services seldom offer palliative care to those dying of MS, despite previous research showing similarities between MS and cancer in the prevalence of palliative-care-related problems, and the need for more palliative care services for pwMS. [22, 23] Even in the general population, it is estimated that 69%-82% of those who die need palliative care, whereas in our study only 4.6% of pwMS died in a hospice, most of whom also had cancer. [24] Randomised controlled trials in pwMS have shown that palliative care can improve patient-reported outcomes, care-giver burden, and are cost-effective. [25–27] Difficulties in determining prognosis can lead to lack of utilisation of end of life care services by those with non-malignant diseases as healthcare professionals may be unsure when to begin the transfer to these services. [28, 29] The use of predictors of progressive dwindling, with a transfer to formative care pathways may improve quality of life for these patients in the progressive stage of their disease, and because of the overlap in techniques, may increase scope for smooth transition into palliative care in the home or hospice environment. [29] An ongoing randomised controlled trial from Solari et al is examining the strengths and limitations of a home-based end-of-life care approach in people with severe MS; this should provide insight into the impact of, for example, home pain management services on MS symptoms and health-related quality of life, an important aspect of formative care. [30]

A qualitative study by Borreani et al. of pwMS, their carers and health professionals, found that adapting to life with disability was of greater importance than end of life care. [31] The interventions proposed in response to their findings implied a formative approach, including domestic support, rehabilitation and psychosocial intervention. [31]

Future work should develop ways to improve the delivery of formative care, assess its acceptability, and whether it could be delivered in complex situations. Input from pwMS and their families is paramount in developing such pathways. Further work could build a quantitative tool to allow clinicians to assess the exact risk of progressive dwindling for an individual patient with a specific combination of risk factors. Such a tool might be utilised to deliver formative care approaches to targeted patient populations, and to evaluate their impact on symptom relief, quality of life, place of death, cost of healthcare, and benefit to carers and family members.

## Conclusion

End of life care has been failing a large number of patients because of uncoordinated services, lack of communication and lack of identification of people who are dying. [3] Formative care, when not properly designated as such can be disjointed and inadequate from the patient and family’s perspective and unnecessarily expensive from a service provider’s angle. [1] This study concludes that these failings might impact particularly upon pwMS, as 73.7% of pwMS followed the progressively dwindling trajectory to death. A marker of these failings lies in the finding that pwMS who progressively dwindle are no less likely to die in hospital than those with other trajectories to death. Ideally, those who progressively dwindle should have lower rates of hospital deaths, as a result of well-timed discussions, adequate planning and well-coordinated formative and palliative care services. This study aimed to identify factors associated with progressive dwindling and found that early disease milestones such as age at onset, progression and wheelchair use can be used as potential predictors, thus allowing timely discussions, a vital step towards providing formative care to those who need it. This study found that 72.5% of pwMS died an MS-related death, and this too was associated with an aggressive early disease course. The use of predictors of progressive dwindling, with a transfer to formative care pathways for years prior to death may improve quality of life for patients in the progressive stage of their disease and their caregivers, and may increase scope for smooth transition into palliative care in the home or hospice environment. We believe that better coordination of medical and social care is paramount in the years prior to death but subsequent to active treatment in pwMS and other populations. The results from this study provide a framework on which to base subsequent carestrategies, and to target those who might benefit most from formative care.

## References

[1] Lynn J, Adamson D. Living Well at the End of Life: Adapting Health Care to Serious Chronic Illness in Old Age Santa Monica, CA: RAND Corporation; 2003 Available from: http://www.rand.org/pubs/white_papers/WP137.html. Also available in print form.

[2] Bowman C, Meyer J. Formative Care: Defining the purpose and clinical practice of care for the frail. J R Soc Med. 2014;107(3):95–8. 10.1177/0141076813512298 . 24334912 PMC3938123

[3] Parliamentary and Health Service Ombudsman. Dying without dignity 2015. Available from: http://www.ombudsman.org.uk/__data/assets/pdf_file/0019/32167/Dying_without_dignity_report.pdf.

[4] Degenhardt A, Ramagopalan SV, Scalfari A, Ebers GC. Clinical prognostic factors in multiple sclerosis: a natural history review. Nat Rev Neurol. 2009;5(12):672–82. 10.1038/nrneurol.2009.178 . 19953117

[5] Koffman J, Gao W, Goddard C, Burman R, Jackson D, Shaw P, et al Progression, Symptoms and Psychosocial Concerns among Those Severely Affected by Multiple Sclerosis: A Mixed-Methods Cross-Sectional Study of Black Caribbean and White British People. Plos One. 2013;8(10). ARTN e75431 10.1371/journal.pone.0075431 WOS:000325434500032. PMC3788806 24098384

[6] Murray SA, Kendall M, Boyd K, Sheikh A. Illness trajectories and palliative care. Brit Med J. 2005;330(7498):1007–11. 10.1136/bmj.330.7498.1007 WOS:000228935100024. 15860828 PMC557152

[7] Reynolds R, Roncaroli F, Nicholas R, Radotra B, Gveric D, Howell O. The neuropathological basis of clinical progression in multiple sclerosis. Acta Neuropathol. 2011;122(2):155–70. 10.1007/s00401-011-0840-0 WOS:000292780500004. 21626034

[8] Pring A. Classification of place of death. Public Health England 2013. Available from: http://www.endoflifecare-intelligence.org.uk/view?rid=762.

[9] Goldacre MJ, Duncan M, Griffith M, Turner MR. Trends in death certification for multiple sclerosis, motor neuron disease, Parkinson's disease and epilepsy in English populations 1979–2006. J Neurol. 2010;257(5):706–15. 10.1007/s00415-009-5392-z . 19946783

[10] Sleeman KE, Ho YK, Verne J, Glickman M, Silber E, Gao W, et al Place of death, and its relation with underlying cause of death, in Parkinson's disease, motor neurone disease, and multiple sclerosis: A population-based study. Palliative Med. 2013;27(9):840–6. 10.1177/0269216313490436 WOS:000324624600007. 23737036

[11] Hirst C, Swingler R, Compston DA, Ben-Shlomo Y, Robertson NP. Survival and cause of death in multiple sclerosis: a prospective population-based study. J Neurol Neurosurg Psychiatry. 2008;79(9):1016–21. 10.1136/jnnp.2007.127332 . 18303108

[12] Scalfari A, Knappertz V, Cutter G, Goodin DS, Ashton R, Ebers GC. Mortality in patients with multiple sclerosis. Neurology. 2013;81(2):184–92. WOS:000330742600017. 10.1212/WNL.0b013e31829a3388 23836941 PMC3770174

[13] Jick SS, Li L, Falcone GJ, Vassilev ZP, Wallander MA. Mortality of patients with multiple sclerosis: a cohort study in UK primary care. J Neurol. 2014;261(8):1508–17. 10.1007/s00415-014-7370-3 WOS:000340052000008. 24838537 PMC4119255

[14] Office for National Statistics. Deaths in England and Wales 2013. Available from: http://www.ons.gov.uk/ons/rel/vsob1/death-reg-sum-tables/2013/info-deaths-2013.html.

[15] Office for National Statistics. Suicides in the United Kingdom 2013. Available from: http://www.ons.gov.uk/ons/rel/subnational-health4/suicides-in-the-united-kingdom/2013-registrations/suicides-in-the-united-kingdom-2013-registrations.html.

[16] National Audit Office Comptroller and Auditor General. End of Life Care: Executive Summary. HC 1043 2008. Available from: https://www.nao.org.uk/wp-content/uploads/2008/11/07081043.pdf.

[17] Gomes B, Calanzani N, Gysels M, Hall S, Higginson IJ. Heterogeneity and changes in preferences for dying at home: a systematic review. Bmc Palliat Care. 2013;12 Artn 7 10.1186/1472-684x-12-7 WOS:000318007900001. PMC3623898 23414145

[18] End of Life Care Strategy—promoting high quality care for all adults at the end of life. Department of Health Report. Report number 9840. 2008.

[19] Rao SM, Leo GJ, Bernardin L, Unverzagt F. Cognitive dysfunction in multiple sclerosis. I. Frequency, patterns, and prediction. Neurology. 1991;41(5):685–91. . 2027484 10.1212/wnl.41.5.685

[20] End of Life Care: Executive Summary. National Audit Office Comptroller and Auditor General. Report number: HC 1043 2008.

[21] Golla H, Mammeas S, Galushko M, Pfaff H, Voltz R. Unmet needs of caregivers of severely affected multiple sclerosis patients: A qualitative study. Palliat Support Care. 2015;13(6):1685–93. 10.1017/S1478951515000607 . 26081132

[22] Moens K, Higginson IJ, Harding R, Impact E. Are There Differences in the Prevalence of Palliative Care-Related Problems in People Living With Advanced Cancer and Eight Non-Cancer Conditions? A Systematic Review. J Pain Symptom Manag. 2014;48(4):660–77. 10.1016/j.jpainsymman.2013.11.009 WOS:000343856500019. 24801658

[23] Galushko M, Golla H, Strupp J, Karbach U, Kaiser C, Ernstmann N, et al Unmet needs of patients feeling severely affected by multiple sclerosis in Germany: a qualitative study. J Palliat Med. 2014;17(3):274–81. 10.1089/jpm.2013.0497 24527993 PMC3952521

[24] Murtagh FE, Bausewein C, Verne J, Groeneveld EI, Kaloki YE, Higginson IJ. How many people need palliative care? A study developing and comparing methods for population-based estimates. Palliat Med. 2014;28(1):49–58. 10.1177/0269216313489367 . 23695827

[25] Edmonds P, Hart S, Gao W, Vivat B, Burman R, Silber E, et al Palliative care for people severely affected by multiple sclerosis: evaluation of a novel palliative care service. Mult Scler J. 2010;16(5):627–36. 10.1177/1352458510364632 WOS:000277837200017. 20305044

[26] Higginson IJ, Costantini M, Silber E, Burman R, Edmonds P. Evaluation of a new model of short-term palliative care for people severely affected with multiple sclerosis: a randomised fast-track trial to test timing of referral and how long the effect is maintained. Postgrad Med J. 2011;87(1033):769–75. 10.1136/postgradmedj-2011-130290 . 21978993

[27] Higginson IJ, McCrone P, Hart SR, Burman R, Silber E, Edmonds PM. Is Short-Term Palliative Care Cost-Effective in Multiple Sclerosis? A Randomized Phase II Trial. J Pain Symptom Manag. 2009;38(6):816–26. 10.1016/j.jpainsymman.2009.07.002 WOS:000279280200002. 19833477

[28] Shipman C, Gysels M, White P, Worth A, Murray SA, Barclay S, et al Improving generalist end of life care: national consultation with practitioners, commissioners, academics, and service user groups. Brit Med J. 2008;337(7674). ARTN a1720 10.1136/bmj.a1720 WOS:000259903300032. PMC2659492 18829640

[29] Strupp J, Romotzky V, Galushko M, Golla H, Voltz R. Palliative Care for Severely Affected Patients with Multiple Sclerosis: When and Why? Results of a Delphi Survey of Health Care Professionals. Journal of Palliative Medicine. 2014;17(10):1128–36. 10.1089/jpm.2013.0667 WOS:000342743200011. 25068391 PMC4195346

[30] Solari A, Giordano A, Grasso MG, Confalonieri P, Patti F, Lugaresi A, et al Home-based palliative approach for people with severe multiple sclerosis and their carers: study protocol for a randomized controlled trial. Trials. 2015;16 ARTN 184 10.1186/s13063-015-0695-0 WOS:000353459400001. PMC4409986 25899519

[31] Borreani C, Bianchi E, Pietrolongo E, Rossi I, Cilia S, Giuntoli M, et al Unmet Needs of People with Severe Multiple Sclerosis and Their Carers: Qualitative Findings for a Home-Based Intervention. Plos One. 2014;9(10). ARTN e109679 10.1371/journal.pone.0109679 WOS:000345743700084. PMC4186842 25286321

